# Round the Hospitals

**Published:** 1920-03-13

**Authors:** 


### ROUND THE HOSPITALS.

At the Investiture held on March 3 at Buckingham Palace, the King personally conferred decorations on the following members of the nursing services:—Royal Red Cross, 1st Class: Miss Mary Newman, Mrs. Mary Sampson, Q.A.I.M.N.S.; Miss Beatrice Tanner, Q.A.I.M.N.S. (R.). Royal Red Cross, 2nd Class: Miss Lilian Swift, R.N.N.S.; Misses May Daly and Mary Furdham, Mrs. Elsie Melville, Misses Mary O'Dowd, Mary Powell, Kate Skinner, and Mildred Stewart,

**ROUND THE HOSPITALS—***(continued)*.

Q.A.I.M.N.S. (R.); Misses Lily Chapman, Amy Martin, Marion McMillan, Gwendoline Quentrall, and Jean Robertson, Mrs. Isabella Storar, T.F.N.S.; Miss Gertrude Piper, C.N.S.; Misses May Francis, Caroline Lawson, and Ellen Munro, B.R.C.S.; Misses Theodora Almack, Margaret Coombes, Mary Earle, Cicely Jackson, and Elizabeth Thompson, V.A.D. All these ladies, together with Miss A. B. Smith, R.R.C., were subsequently received by Queen Alexandra at Marlborough House.

At a second Investiture held at Buckingham Palace on March 5 the King personally conferred decorations as follows:—Bar to the Royal Red Cross: Miss Agnes Weir, Q.A.I.M.N.S.; Royal Red Cross and Bar: Miss Catherine Stronach, Q.A.I.M.N.S.; Royal Red Cross, 1st Class: Misses Mary Cooper, Ethel Devenish-Meares (also to receive the Military Medal), and Lilian Wheatley. Royal Red Cross, 2nd Class: Miss Olive Matthews, Q.A.I.M.N.S.; Misses Florence Clieve, Winifred Hoare, Florence Morgan, Jessie Scott, and Annie Wright, Q.A.I.M.N.S. (R.); Misses Gertrude Chandler, Lilian Clieve, and Mary Stollard, T.F.N.S.; Misses Gertrude Male, B.R.C.S.; Miss Mary Mathwin, C. & W. Hospitals; Miss Annie Martin, S.A.M.N.S. The King conferred the Military Medal on Miss Rosa Brain, T.F.N.S. The above, with Dame Sidney Browne, R.R.C., were subsequently received at Marlborough House by Queen Alexandra.

H.M. Queen Alexandra has graciously consented to unveil Sir George Frampton's statue forming the Nurse Cavell Memorial at the end of St. Martin's Lane. The ceremony will take place on Wednesday, March 17. Many notable people have signified their intention to be present or represented, including delegates from Belgium.

An interesting relic of Miss Edith Cavell has been discovered among the records of the London County Council. It is an application dated October 8, 1902, by Miss Edith Louisa Cavell for appointment as assistant superintendent of Farmfield Reformatory. With the application are included three pages of testimonials, copied in Miss Cavell's writing. The date of her birth is given as December 4, 1865, and this date will be engraved on Sir George Frampton's statue, instead of 1866 erroneously given previously. The document is to be on view in the lobby to the L.C.C. Chambers.

Lord Knutsford gave some astonishing examples of the increased cost of living at the recent meeting of the Court of Governors of the London Hospital. They throw great light on the causes which are plunging all the voluntary hospitals into difficulties. In the decade from 1908 to 1918 expenditure has risen from £110,000 to £200,000. The cost of the nursing has enormously increased. Every article has undergone a fabulous rise in price. Lord Knutsford alluded to their recent purchase of 1,500 cotton sheets at 19s. 6d. each. Before the war they could get linen ones at 5s. each. The scheme for making a charge of 10s. a week each to patients to meet part of the cost of food is estimated to bring in £10,000, and will shortly be carried into effect.

Speaking at a drawing-room meeting, held on Monday afternoon, 8th inst., at "Beechwood," Hampstead Lane, Highgate, in connection with the Ladies' Association of the Great Northern Central Hospital, Holloway, Lady Sargant, R.R.C. (Chairman of the Ladies' Association), stated that many years ago the Association had built the outpatient department and the children's ward, and it contributed regularly to the Maintenance, Samaritan, Endowment, and Convalescent Home funds. She appealed for new members to help in the work, which was increasing, and thirty-eight ladies were enrolled. Mr. G. B. Mower White, F.R.C.S., said that, in the course of a few years, the hospital, which was bound to become the most important in North London, had been called upon to treat 50,000 additional out-patients.

The death is reported at Musselburgh, on the Firth of Forth, of Miss A. L. Pringle, one of Miss Florence Nightingale's closest friends and an early pupil in the Nightingale Home at St. Thomas's. Her passing recalls a notable chapter of nursing history brought vividly to mind by Sir James Affleck, M.D., LL.D., in the *Scotsman* of March 2. It was in the early seventies, when the building of the new Edinburgh Infirmary had begun, that Miss Pringle was selected, at the age of 27, to fill the post of superintendent of nurses at this famous institution, with a view to a complete re-organisation of the nursing and domestic arrangements. The numerous difficulties involved in changing the system and replacing old and tried workers by trained women were met by Miss Pringle with a skill, tact, and kindness which won the admiration of all. When the new infirmary was ready the business of transferring many hundreds of patients with the working staff was accomplished by Miss Pringle, working in co-operation with Surgeon-General Fasson, the late superintendent, in a marvellously rapid, complete and comfortable manner. The new sphere afforded Miss Pringle unrivalled opportunities for developing the work of training nurses which lay nearest her heart. Her mental powers were of a high order and she possessed in marked degree those qualities of good sense, sympathy, discernment of character, and firmness united with gentleness which are characteristic of all great matrons. She remained fifteen years in the service of the infirmary.

Miss Pringle's connection with St. Thomas's Hospital was more brief. She was persuaded by Miss Nightingale, with whom she enjoyed unbroken friendship dating from her pupil days, to leave the Royal Infirmary and return to St. Thomas's as matron. She entered upon her new post with high promise and her rule there, as at Edinburgh, was

**ROUND THE HOSPITALS**—(*continued*).

marked by successful reforms. She resigned her post, however, after a comparatively short term of office, on entering the Roman Church. She subsequently filled other posts at home and abroad with conspicuous ability, and gave her services even during the late war in a soldiers' hostel until her health proved unequal to such tasks. Her chief life-work was in laying the foundation of the Edinburgh Royal Infirmary Nurses' Training-School

The *Daily News* has opened its columns to a discussion on the "hard case of the nurse." It is a pity that they do not scrutinise more closely some of the statements made. One nurse, for instance, who belongs to a Nurses' Co-operation "which provides her with work and permits her to take her fees of £3 3s. a week from her patients," complains that she has to pay a percentage to the Co-operation and keep herself in *food, lodging*, clothes and uniform. This is manifestly misleading. All private nurses are boarded or lodged by their patients or provided with an allowance if they have to sleep out. On £3 3s. a week, plus board and lodging, any woman can live very well, take a good holiday, and put by for the future. There are plenty of "hard cases" among nurses, but this is not one of them.

We learn that it has not yet been possible to put into practice the forty-eight-hour week at St. George's Hospital. The principle has been agreed to by the hospital authorities, as announced in our issue of 28th ultimo, and as soon as the nurses necessary to increase the staff to the required extent have been engaged, the forty-eight-hour week will immediately come into force. At present seven of the twenty nurses are appointed. Further applications are being dealt with, and it is hoped that the remaining thirteen nurses may be appointed within the next week or two.

The *Daily Telegraph* Fund for Nurses is approaching the completion of its second hundred thousand shillings, the total as we go to press being 194,646 shillings. Hospital nursing staffs and members of the College of Nursing continue to work hard in the good cause. We note another contribution from the Edinburgh Centre of the College, making their total up to 1,342 shillings; also further instalments from the Nightingale Training School, the Royal Hampshire County Hospital, as well as from the Dundee and London Centres of the College. The Royal Infirmary, Glasgow, and the Dreadnought at Greenwich, send contributions. If the Fund mounts up slowly it must be remembered that money flows in a slender trickle just now for all good causes, possibly because they are numerous, but also for other causes. A writer in *The Times* complains that at the present moment no prices are too extravagant for the buyers but no sum is too small for the subscription. The writer cites the case of the London Fever Hospital, which recently appealed for £50,000. The Press gave the appeal the most complete publicity; the Lord Mayor gave a reception at the Mansion House; Princess Mary lent her name to the appeal and came herself to the City. The response was 10 per cent.—£5,000—from which has to be deducted £1,400 for expenses. We mention this fact to show what hard work is required to obtain money for the most necessary purposes. The "new rich" have not yet learned their duty, and to teach it them requires perseverance and faith.

The inquest on the body of Miss Florence Nightingale Shore, aged fifty-five, the nursing sister who was found unconscious with severe injuries in the London-Hastings express, was concluded at Hastings on March 3. A verdict of "Murder by some person or persons unknown" was returned, and the police appear to have lost all hope of tracing the assailant. Death was caused by coma due to fracture of the skull and injury to the brain, such as might have been caused by the butt end or side of a revolver. Ten persons have been detained in connection with this case, and over a hundred inquiries made, but with no result. Efforts are being made to found a permanent memorial to Miss Shore, which is to take the form of a new home in connection with the work of the Hammersmith District Nursing Association. Its former headquarters, Carnforth Lodge, where Miss Shore often stayed, has been sold. If the friends of the deceased nurse, with the aid of the public, can raise £5,000, a new centre could be formed for the treatment of children, with a clinic for disabled men, and here the Queen's nurses could have their quarters. It is a very happy part of this plan to reserve one room in which to keep tangible mementoes of Miss Shore's life work, to be made available as a guest-room for Queen's nurses passing through London. Contributions of 1s. or upwards may be sent to the Hon. Treasurer, F.N.S. Memorial, Mr. Marshall Hays, 32 St. Peter's Square, Hammersmith, W. 6.

Another case of assault, this time on a V.A.D. nurse employed at the Connaught Hospital, Aldershot, illustrates the spirit of lawlessness abroad. It used to be an unheard-of thing for a nurse to be in any way molested, and, however lonely the road, nurses have been accustomed to feel complete confidence in the protection afforded by their uniform. Miss Perry travelled down from Waterloo to the North Camp Station with several soldiers in her compartment. She was attacked later on, about 10 p.m., by one of her fellow-passengers, a "tall, dark, smart-looking soldier," who struck her in the face with his stick. She remembers nothing after receiving the blow, and is now lying in a serious condition from shock, with cuts and bruises on her face, in the Canbridge Hospital, Aldershot.

We learn from the *American Journal of Nursing* for February that eight hospitals in New York City are experimenting with the eight-hour day or a fifty-two-hour week. No two are organised quite on the same lines. In some cases, it is

**ROUND THE HOSPITALS**—(*continued*).

stated, the change has been made with no increase of staff and in others with a very slight addition, three to ten per cent. In one hospital a group of affiliating nurses has supplied the extra hands; in another extra pupils; in a third fully trained nurses have been employed. One hospital carries a straight eight-hour shift, 7 to 3, 3 to 11, 11 to 7, with one full afternoon a week off. Another has the straight eight-hour night from 11 to 7, but the day service is in broken periods. In another the night nurses come on at 7 and stay through the night, and extra time is given night nurses at the end of their period of night duty so as to maintain the fifty-two hour week. Two of the schools have an eight-hour day and a ten-hour night with extra allowance at the end of the night period. It is interesting to learn that the changes have caused no annoyance to the patients, while the nurses are better in health and fresher for their class work. An acute shortage of candidates is afflicting the training-schools, as well as of helpers or attendants.

---

The committee of the West End Hospital, Welbeck Street, are to be congratulated on the happy thought which has prompted the presentation to their matron, Miss Catharine E. A. Thorpe, A.R.R.C. On Friday, February 27, Miss Thorpe was summoned to the committee meeting, and there presented by the Vice-Chairman with an illuminated note on vellum, stating that "The members of the staff and other friends desire to offer you the accompanying purse in grateful appreciation of your long and devoted service to the West End Hospital." Then followed the names of the Chairman, Treasurer, Secretary, and a number of members of the committee and the medical staff, together with a few of the principal supporters of the hospital. The purse contained a cheque. Miss Thorpe, who was trained at the London Hospital, and was later sister at the Metropolitan Hospital, has been for sixteen years matron of the West End Hospital. She has seen service both in the South African war and the Great War, and was at Mons.

---

An urgent appeal has been made to the British Committee of the Russian Red Cross to create a base at Constantinople for relieving Russian civilian refugees congregated there. A complete Red Cross unit has gone forward to prepare this base, and has decided to equip a hospital of 150 beds, which include fifty for children, with an entire British staff.

---

A final balance sheet has been issued by Dr. Barnes of the Cumberland Branch of the B.R.C.S., showing how the funds remaining in hand, £10,698, have been distributed. Numerous donations have been made to nursing associations in the county, including £915 to the Cumberland N. A. and £105 to the Carlisle District N. A. In addition to these welcome sums the nursing associations benefit to the extent of £3,000 by a further allocation from the Joint Finance Committee of the British Red Cross and the Order of St. John. Dr. Barnes is now retiring from the joint office of Hon. Secretary and Treasurer to which he has devoted so much time and energy. His efficiency in office may be estimated from the fact that the total "administration expenses" of allocating the sums named have amounted to merely £17.

---

A very pitiful fraud on a nurse was exposed at the Central Criminal Court when Percy Miller, contractor, pleaded "Guilty" to obtaining money from Edith Cooke by false pretences. The defendant had been a private in the R.A.S.C., and was received as a patient at the Greenwich Infirmary, where Mrs. Cooke was at that time a nurse. He represented himself to be a member of the Stock Exchange, and on this understanding she entrusted all her savings to him, amounting to £857. It was proved that he had played the same part with another lady, a Miss Otto. It was stated in his defence that he was a man of good family, and that he had been a member of a provincial stock exchange. Miss Otto had already received back her money, and that of Mrs. Cooke was in Court, and would be paid over to her. In these circumstances the Recorder postponed judgment till the next session. It has been a narrow escape for both ladies.

---

The extraordinary case of Helen Aileen Sinclair, aged twenty-eight, who has been convicted at Durham Assizes of obtaining goods under false pretences as a trained nurse, shows once more the extent to which the nursing profession is made to serve as cloak for evil doings. The prisoner has been three times charged with bigamy, and is described by an Edinburgh detective as the most notorious female criminal he had encountered during twenty-four years' experience. Her link with the nursing profession appears to be as shadowy as that with the Edinburgh University, of which she posed as a graduate. She has been sentenced to three years' penal servitude with five years' preventive detention, a drastic sentence. She seems to have one of those dangerous neurotic dispositions which experience exhilaration from incendiarism, for her presence has more than once been the signal for unaccountable outbreaks of fire. Is penal servitude the right way to deal with this type of case?

---

In times of emergency the Metropolitan Asylums Board engages nurses from various nursing institutions to fill vacancies at infectious hospitals, paying the institution fees from $1\frac{1}{2}$ to $2\frac{1}{2}$ guineas per week per nurse. It happens at times that these nurses contract infectious disease and in such cases the Board has hitherto ceased payment. The remuneration of the nurses under such circumstances is a heavy expense to the nursing institutions, and they have now appealed for some concession. The Asylums Board cordially admits that the nursing organisations have afforded great assistance at very critical times, and now proposes to pay fees as usual in cases where nurses contract disease whilst employed at hospitals. This seems to us an entirely reasonable decision.

